# Borderline shades: Morphometric features predict borderline personality traits but not histrionic traits

**DOI:** 10.1016/j.nicl.2023.103530

**Published:** 2023-10-14

**Authors:** Miriam Langerbeck, Teresa Baggio, Irene Messina, Salil Bhat, Alessandro Grecucci

**Affiliations:** a Faculty of Psychology and Neuroscience (FPN), Maastricht University, Netherlands; b Department of Psychology and Cognitive Sciences (DiPSCo), University of Trento, Italy; c Universitas Mercatorum, Rome, Italy; d Department of Cognitive Neuroscience, Faculty of Psychology and Cognitive Neuroscience (FPN), Maastricht University, Netherlands; e Centre for Medical Sciences (CISMed), University of Trento, Italy

**Keywords:** Borderline, Personality disorder, Histrionic, Personality traits, Machine learning, Kernel Ridge Regression

## Abstract

Borderline personality disorder (BPD) is one of the most diagnosed disorders in clinical settings. Besides the fully diagnosed disorder, borderline personality traits (BPT) are quite common in the general population. Prior studies have investigated the neural correlates of BPD but not of BPT. This paper investigates the neural correlates of BPT in a subclinical population using a supervised machine learning method known as Kernel Ridge Regression (KRR) to build predictive models. Additionally, we want to determine whether the same brain areas involved in BPD are also involved in subclinical BPT. Recent attempts to characterize the specific role of resting state-derived macro networks in BPD have highlighted the role of the default mode network. However, it is not known if this extends to the subclinical population. Finally, we wanted to test the hypothesis that the same circuitry that predicts BPT can also predict histrionic personality traits. Histrionic personality is sometimes considered a milder form of BPD, and making a differential diagnosis between the two may be difficult. For the first time KRR was applied to structural images of 135 individuals to predict BPT, based on the whole brain, on a circuit previously found to correctly classify BPD, and on the five macro-networks. At a whole brain level, results show that frontal and parietal regions, as well as the Heschl’s area, the thalamus, the cingulum, and the insula, are able to predict borderline traits. BPT predictions increase when considering only the regions limited to the brain circuit derived from a study on BPD, confirming a certain overlap in brain structure between subclinical and clinical samples. Of all the five macro networks, only the DMN successfully predicts BPD, confirming previous observations on its role in the BPD. Histrionic traits could not be predicted by the BPT circuit. The results have implications for the diagnosis of BPD and a dimensional model of personality.

## Highlights

• Neural correlates of borderline personality traits (BPT) are investigated in a subclinical sample using a supervised machine learning approach.
• Some of the same brain areas that predict borderline personality disorder also predict BPT, including the Heschl’s area, the thalamus, the cingulum, and the insula.
• BPT predictions increase when considering only the regions limited to the brain circuit derived from a study on BPD, confirming a certain overlap in brain structure between subclinical and clinical samples.
• Of all the five macro networks, only the DMN successfully predicts BPD, confirming previous observation on its role in the BPD.
• Histrionic traits could not be predicted by the BPT circuit. The results have implications for the diagnosis of BPD and a dimensional model of personality.

## 1 Introduction

Borderline personality disorder (BPD) is a recognized category of personality disorders listed in the Diagnostic and Statistical Manual of Mental Disorders (DSM-5) (American Psychiatric Association, 2013), affecting around 1.6 % of the general population (Mendez-Miller et al., 2022) and thus resulting as the most diagnosed personality disorder (Trull et al., 2010). The diagnosis of BPD relies on observable behavior and is characterized by an unstable pattern of affective regulation, impulsive control, and interpersonal relationships (Dadomo et al., 2016, Dadomo et al., 2018, Dadomo et al., 2022, De Panfilis et al., 2019, Grecucci et al., 2022), that could include impulsive aggressive outbursts, health-sabotaging-behavior (i.e., cutting or manipulation on injuries) or persisting suicidal inclinations (Mendez-Miller et al., 2022). As these symptoms are presented in a strongly fluctuating picture within patients (Davies et al., 2020) a clear psychiatric diagnosis is often complex and difficult (Grecucci et al., 2022, Rao et al., 2020).

Regarding the diagnosis, the DSM, and the International Classification of Diseases (ICD) are the two systems currently used to diagnose a patient with a mental disorder. The categorical approach was applied to distinguish between different personality disorders until the latest versions of the manuals. It included 10 different personality disorders, organized into three clusters. However, these diagnostic categories show an excessive overlap in symptoms, which limits the diagnostic function and goodness of fit (Siefert et al., 2022). Addressing this issue of comorbidity between PDs, the current DSM-5 and ICD-11 suggest different approaches: a hybrid system and a purely dimensional categorization of personality traits. Personality traits are identified on a spectrum, usually using five domains of personality (Ashton, 2017). Within this hybrid model, the categories have been reduced from ten to six, excluding histrionic personality disorder (HPD). This proposed model is to be found in section III of the DSM-5, offering an approach to a new guideline but intending to support the diagnosis via the former categorical system (American Psychiatric Association, 2013). The ICD-11 argues instead that since personality traits are defined on a spectrum, this also applies to personality disorders (Tyrer et al., 2015) and presents a fully dimensional classification using trait-based personality features.

To overcome the limitations of the current diagnostic systems, which are based on observable signs, affective neuroscience has emerged as a developing field. The discovery of neurobiological markers for specific psychopathological conditions can serve as an objective tool for the diagnosis of PD (Grecucci et al., 2022). Neuroimaging studies on BPD have revealed crucial information about its neural correlates. For example, previous studies have found brain changes in the thalamus (Nenadić et al., 2020, Prossin et al., 2010, Xu et al., 2016), the amygdala (Minzenberg et al., 2008, Ruocco et al., 2012, Schulze et al., 2016), and fusiform region in patients with BPD (Bertsch et al., 2019).

Other studies have focused on specific networks involvement in BPD, investigating the “triple network model”. According to this model, borderline patients show functional impairment in the default mode network (DMN), the salience network (SN), and the central executive network (CEN) (Doll et al., 2013). The DMN is activated during rest and consists of two major hubs, corresponding to the posterior cingulate cortex (PCC) and the medial prefrontal cortex. It also includes areas like the hippocampus and the cingulate gyrus, which are known to play a role in semantic memories, linked to internal thought, while medial prefrontal areas are related to social cognition as well as self-monitoring and emotion regulation (Menon, 2011). The SN is comprised of the anterior cingulate cortex and the fronto-insular cortex, as well as subcortical structures containing the amygdala and the ventral tegmental area. It is associated with the detection and integration of relevant stimuli, along with the selection of an appropriate behavioral response and the switch between the DMN and the CEN. Furthermore, the subcortical areas are included in emotion and reward processing (Menon, 2011, Quattrini et al., 2022). The core parts of the CEN are the dorsolateral prefrontal cortex (dlPFC) and the lateral posterior parietal cortex (PPC). It is important for executive control during goal-directed behavior and for maintaining and manipulating information in working memory when performing a task that requires attention (Menon, 2011, Quattrini et al., 2022). A recent study by Quattrini et al. (2022) investigated the possibility of structural changes in the “triple network” in borderline patients. By using diffusion tensor imaging (DTI) techniques the authors found white matter (WM) alterations in the form of structural connectivity in all three networks. According to the authors, this result could indicate a reduced myeline development, which could be caused by an impaired maturation process (Quattrini et al., 2022).

In the above cited studies borderline personality disorder was classified according to the categorical approach, e.g. comparing patients against healthy controls. Although these results support the diagnostic categorical approach, no previous study used a dimensional approach to understand the neural bases of personality disorders. We believe affective neuroscience can significantly help us in understanding personality from both the perspective of categories and dimensions.

Despite the progresses in categorical understanding, previous studies suffered from some methodological limitations. First, the use of mass univariate analysis allows only to look at each voxel separately, and therefore it ignores statistical relationships between voxels (Lapomarda et al., 2021, Sorella et al., 2019). Second, only the average of individuals within each group was considered, thus ignoring individual variances. In addition, some studies used a region of interest (ROI) approach, limiting the results to a small number of previously defined regions, instead of whole-brain strategies (Pappaianni et al., 2019, Saviola et al., 2020, Sorella et al., 2019). Finally, the generalizability of these findings has not been tested to new cases.

To overcome those limitations the use of machine learning (ML), an artificial intelligence method, also known as multi-voxel pattern analysis in neuroscience, represents an alternative approach. It has been shown to be very effective in the diagnostic classification of individuals and for differentiating patients from healthy controls, based on MRI. ML has been demonstrated to increase the sensitivity of brain imaging by considering the information contained in a distributed spatial pattern of brain activity rather than just a single voxel or location (Norman et al., 2006). Furthermore, because ML examines patterns that best distinguish healthy controls from, for example, BPD, it is able to categorize a person belonging to one of the two groups in a new data set, and create individual predictions that can be applied in the clinical setting (Rondina et al., 2018, Vieira et al., 2020). The Kernel Ridge Regression (KRR) approach offers the added advantage of enabling the identification of brain areas contributing to the model as the most relevant sources for the classification, based on whole-brain pattern-based information (Mourao-Miranda et al., 2012). The development of predictive models is important because they can serve as biomarkers for computerized objective diagnosis tools by identifying brain features associated with pathological characteristics (Grecucci et al., 2022, Vai et al., 2020). Additionally, they might also help to clarify the scientific accuracy of one of the proposed diagnostic systems for personality disorders. With the use of supervised Kernel machine learning on MRI data, Grecucci and colleagues (2022) were able to construct a predictive model for BPD and outlined a specific neuronal circuit, including the right Putamen, the left thalamus, the right fusiform gyrus, the right amygdala, the lingual gyrus, the right middle, and superior orbitofrontal cortex (OFC), the left pallidum, the left fusiform gyrus, and portions of the cerebellum. These findings were supported and extended by another study from Grecucci et al. (2023). In this study, a combination of unsupervised and supervised machine learning was applied to first parcel the brain into networks of covarying gray and white matter concentration and then to build a predictive model able to classify individuals with BPD. They outlined similar areas that are predictive of BPD including the post-central and precentral gyri, the insula, the superior, middle and inferior frontal gyrus, the parietal lobule, the amygdala, cerebellar portions, and the hippocampus (Grecucci et al., 2023). Although these studies are, to our knowledge, some of the first using ML to outline a distinct brain circuit for BPD they come with certain limitations. First, the numerosity of the sample size (20 BPD participants against 45 healthy controls) limits the generalization of the results. Second, a categorical rather than a dimensional approach was used to select the clinical group, thus excluding the possibility to understand and detect the individual differences that characterize such personalities. Additionally, the possible neural effect of the usage of psychotropic drugs, and psychotherapic treatments could not be excluded.

In light of these limitations, the first aim of this paper is to use a dimensional approach to the study of personality, by considering BPT in a subclinical population. To do that, a supervised machine learning method will be used to build a predictive model of BPT that captures individual differences and allows generalization. We hypothesize that a brain circuit predicting borderline traits may include some of the brain structures previously identified in BPD, including the amygdala and the thalamus, which are associated with emotional dysregulation and impulsivity in patients with BPD (Grecucci et al., 2023, Grecucci et al., 2022, Lapomarda et al., 2021). Moreover, we predict the OFC and the cerebellum to be involved in the trait circuit, as they are linked to affective disturbance and deficiency in emotional control.

If a continuum of personality traits between subclinical and clinical populations does exist, then it should be possible in principle to find at least some brain regions similarly affected. In other words, the same circuit that is implicated in BPD may also be involved in borderline traits, and if this is the case, it may be possible to predict borderline traits based on this circuit. To test this hypothesis, we will try to predict BPT in our subclinical sample by considering only the regions of the circuit previously found to correctly classify BPD compared to healthy controls (HC) (Grecucci et al., 2022). If this prediction is possible, this may be interpreted as further proof of a continuum between BPD and BPT. Thus, the second aim of the present study is to test the possibility of predicting BPT by using the network that classifies BPD from HC. We hypothesize that this is the case and that this BPD circuit prediction (Aim 2) outperforms the whole brain prediction (Aim 1) because it relies on the most important regions and at the same time excludes the irrelevant regions from the predictive model.

One additional hypothesis we want to test is whether the abnormalities in GM associated with borderline traits can be ascribed to one of the specific macro-networks or not. A recent study has indeed found that borderline patients show functional impairment in the so-called triple network (default mode network (DMN), salience network (SN), and central executive network (CEN)) (Doll et al., 2013). Since it has been shown that resting state macro-networks are also present at a structural level (Baggio et al., 2023, Grecucci et al., 2022, Meier et al., 2016, Vanasse et al., 2021), we want to test the hypothesis that macro-networks abnormalities characterize also BPT (in a subclinical population). Thus, the third aim of the paper is to build a predictive model of borderline traits based on previously defined macro-networks, that are known to show dysfunctions in a wide range of psychiatric diseases. We hypothesize that all the networks included in the triple network model e.g. Default mode network, Salience network, and Executive network, but not the Visual and Sensorimotor networks, can predict borderline traits. Of all these networks, we also predict that the DMN should be the most important in predicting BPT.

Last but not least, another intriguing hypothesis we want to test in the present study concerns the possible overlap between borderline and histrionic traits. Borderline traits display a certain overlap with HPD (Kaess et al., 2013). Indeed, according to the DSM-5, the criteria for HPD include a pattern of excessive emotion (i.e., rapidly shifting, or exaggerated outbursts) and attention-seeking behavior (i.e., drawing attention by use of physical appearance) (American Psychiatric Association, 2013), which resemble the criteria of BPD. Previous studies have tried to classify BPD and HPD into subtypes, showing that both disorders can be categorized in at least one subtype that displays symptoms of the other disorder (Blagov and Westen, 2008, Smits et al., 2017). Partial overlap of symptoms could be explained by a common genetic predisposition accounting for affect regulation, impulsivity, and self-cohesion (Bakkevig and Karterud, 2010, Kendler et al., 2008). Self-cohesion consists of the person's subjective experience of having a unified and stable sense of self (Palombo, 1992). Due to these findings and the limited amount of research on HPD (Zarnowski et al., 2021), it remains difficult to distinguish HPD and BPD from each other, and thus confusion in diagnosing remains (Blagov & Westen, 2008). To clarify the eventual neural overlap between borderline and histrionic personalities, we want to test the hypothesis according to which the same circuits predicting borderline traits (derived from Aim 1) can successfully predict the histrionic traits in the same population (Aim 4). One possibility is that our model can predict HP traits from the BD traits’ circuit found in Aim 1. If this is true, it may be due to some shared neural basis between BP and HP. However, if the model fails to predict HP traits from the BP traits circuit, it might be interpreted as the two personalities having distinct neural bases.

## 2 Methods

### 2.1 Participants

The structural MRI data were taken from the MPI-Leipzig Mind Brain-Body dataset (OpenNeuro database, Accession Number: ds000221) (Babayan et al., 2020) which includes MRI and behavioral data of 318 participants. The participants took part in one or two projects (LEMON protocol and Neuroanatomy & Connectivity Protocol) conducted by the Max Planck Institute (MPI) of Human Cognitive and Brain Sciences in Leipzig. The project was authorized by the ethics committee of the University of Leipzig (154/13-ff) (Babayan et al., 2019). For our analysis, we selected the data from 135 healthy participants (F = 64, M = 71, age = 31.94±15.06) from the Neuroanatomy & Connectivity Protocol (N&C). Inclusion criteria for our study were based on good health, no intake of medication, and no history of substance abuse or neurological diseases (e.g., epilepsy or Alzheimer’s). Additionally, the availability of the personality style and disorder inventory (PSSI) questionnaire scores was a necessary inclusion criterion. The PSSI, developed by Kuhl and Kazén in 1997, is a self-report instrument assessing the relative expression of personality traits formulated as non-pathological counterparts to the personality disorders described in the DSM-IV and ICD-10 psychiatric diagnostic manuals. The “impulsive-borderline” (BL) and “agreeable-histrionic” (HI) subscales of the PSSI were used in this study. Participants rated each item on a 4-point Likert scale (from 0 to 3), and the sum of the ratings for the 10 items belonging to a given scale was calculated as a continuous scale score. The PSSI has shown satisfactory to good reliability and validity coefficients (Kuhl & Kazén, 1997). Participants’ demographic and behavioral data are reported in Table 1.

**Table 1:** Demographics and PSSI scores.

Demographics	
Participants (N)	135
Age (years)	32.90 ± 16.43
Sex	64 females; 71 males

PSSI subscales	
Borderline	6.86 ± 4.14
Histrionic	12.92 ± 4.40

### 2.2 MRI data

The MPI-Leipzig Mind Brain-Body dataset includes quantitative T1-weighted, functional, resting state, and diffusion-weighted images which were collected at the Day Clinic for Cognitive Neurology of the University Clinic Leipzig and the Max Planck Institute for Human and Cognitive and Brain Sciences (MPI CBS) in Leipzig, Germany (Babayan et al., 2020). For our study, we considered only the T1-weighted images. Magnetic Resonance Imaging (MRI) was performed on a 3T Siemens MAGNETOM Verio scanner (Siemens Healthcare GmbH, Erlangen, Germany) with a 32-channel head coil. The MP2RAGE sequence consisted of the following parameters: sagittal acquisition orientation, one 3D volume with 176 slices, TR = 5000 ms, TE = 2.92 ms, TI1 = 700 ms, TI2 = 2500 ms, FA1 = 4°, FA2 = 5°, pre-scan normalization, echo spacing = 6.9 ms, bandwidth = 240 Hz/pixel, FOV = 256 mm, voxel size = 1 mm isotropic, GRAPPA acceleration factor 3, slice order = interleaved, duration = 8 min 22 s.

### 2.3 Preprocessing

Preprocessing was performed on all the anatomical images using SPM12 and the Computational Anatomy Toolbox (CAT12), in the MATLAB environment. First, a manual re-orientation through the anterior commissure was performed. Images were segmented into gray matter, white matter, and cerebrospinal fluid. This study focused only on gray matter for the following steps. To normalize each subject’s gray matter image to the average DARTEL template and the Montreal Neurological Institute (MNI) space, a diffeomorphic anatomical registration exponential Lie algebra (DARTEL) approach was applied. Lastly, a smoothing of 10 was applied (Monté-Rubio et al., 2018).

### 2.4 Machine learning-based prediction

**Fig. 1:**
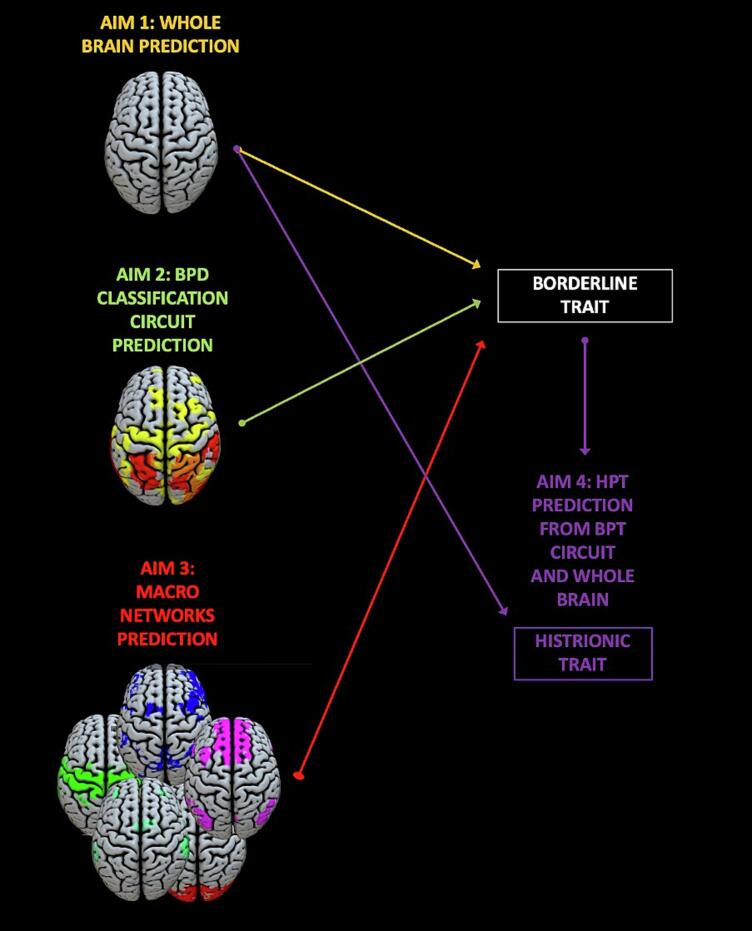
Schematic workflow of the analyses. Analyses were separately conducted for borderline and histrionic traits. For the borderline traits, we ran three analyses: Aim 1: Whole brain prediction; Aim 2 BPD classification circuit of Grecucci et al., 2022 prediction; Aim 3: Macro networks prediction. For what concerns the HP traits we tested the hypothesis that the network predictive of BP traits (aim 1) and whole brain could predict the HP traits (Aim 4).

**Fig. 2:**
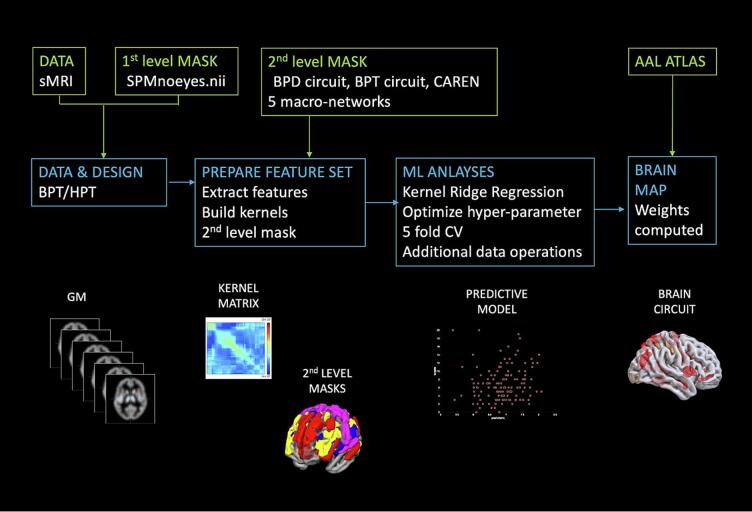
Outline of the schematic workflow of the machine learning process in the PRonTO toolbox. The five analysis modules show the steps that were used in the study. Figure adapted after (Schrouff et al., 2013).

Kernel Ridge Regression (KRR) was used inside the PRoNTO toolbox (*Pattern Recognition for Neuroimaging Toolbox (*PRoNTo, n.d*)*) in Matlab (The MathWorks Inc., 2022). The primary objective of (machine) learning is the generalization to unseen data points, which includes the capacity to forecast a corresponding data point from a given input set. Real-world data often require nonlinear approaches to uncover correlations that allow prediction of the properties of interest. Using KRR, real-world data are converted to a linear estimate before performing regression on the data. (He et al., 2014, Hofmann et al., 2008). Ridge regression is used in situations where the independent variables are highly correlated because it reduces the multicollinearity problem by using a tolerable degree of a biased estimator to archive a smaller Mean Square Error (Kibria & Banik, 2020). Previous neuroimaging studies have shown that KRR has a high estimation prediction level and is, therefore, an accurate method for predictions (Chu et al., 2011). Additionally, it is a fast and computationally effective method for MRI parameter estimation (Nataraj et al., 2017), which enables us to generate a predictive model of borderline/histrionic traits. The present study focused on several predictive models. For all models, only gray matter features were included and covered with a general no-eyes mask (SPMnoeyes.nii), which is used to eliminate any features that are not of interest (i.e. voxel outside of the brain), to optimize the feature set steps (Ashburner et al., 2018). A KRR was then applied, with a k-fold nested cross-validation (CV) model (Schrouff et al., 2013). Cross-validation is used to evaluate a model’s generalizability and to secure that the data are not overfitted by the model. The data is divided into a training set, for training the model (e.g., fit parameters) and into a testing set, for evaluating the model’s performance on unused data. An approximately unbiased estimator of the real generalization error of the model is created by repeatedly splitting the data in this way. In our study, the data was divided into k (number of folds) = 5 (Rodriguez et al., 2009), meaning 20 % of the data was used to test the model and 80 % to train it. An optimized hyper-parameter tuning (0.0001, 0.01, 1, 10, 100, 1000) was used to obtain the nested CV (Dadomo et al., 2022). Using a nested CV can lead to improved results (Claesen & De Moor, 2015). To further normalize the distribution, mean center features and normalized samples were chosen. To avoid confounding, the effect of sex was regressed out from the model. In order to estimate the performance of the model and the significance of the results, we used 3000 permutations. Pearson’s correlation coefficient, the goodness of prediction (R2), mean squared error (MSE), normalized mean squared error (nMSE), and their relative p values were used to evaluate the goodness of the model in predicting new cases. For all aims this procedure was applied. In addition to this, for aims 2 and 3 we used, second-level masks, the BPD mask derived from Grecucci et al. (2022) (Aim 2), and the five macro-network masks (Default mode network, Salience network, Central Executive network, Sensory network, and Visual network) derived from CAREN macron networks atlas (Doucet et al., 2019) (Aim 3). In the latter case, a Bonferroni correction for multiple comparisons was administered to adjust the significance level (α = 0.05/5 comparisons = 0.01). To test the hypothesis of Aim 4 (overlap between the two traits), the circuit derived from the BP traits (Aim 1) was used as a second-level mask on the model testing for histrionic traits. See Fig. 1 for a schematic overview. Fig. 2.

## 3 Results

### 3.1 A predictive model of borderline traits based on whole brain features

**Fig. 3:**
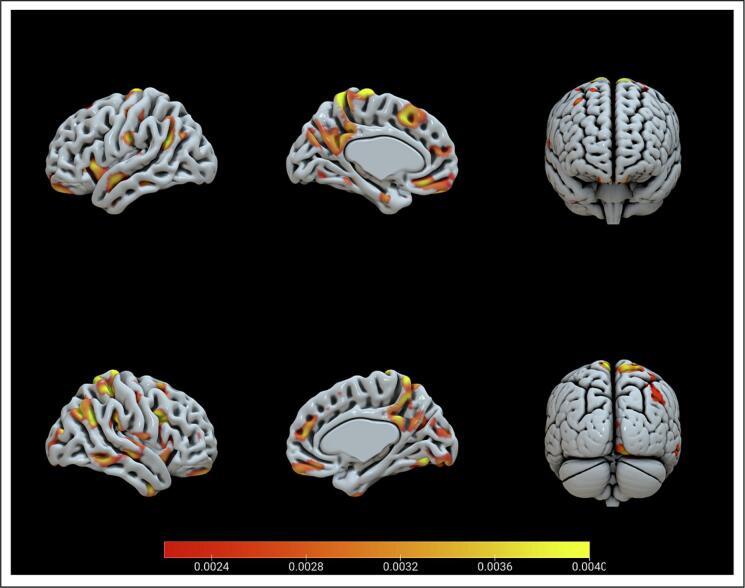
Results for significant brain regions predicting borderline trait. Multiple Kernel Learning regression of gray matter on borderline traits. Surface plots, including subcortical regions.

Whole brain gray matter features significantly predicted borderline traits (r = 0.11p = 0.037; R2 = 0.01p = 0.6328; MSE = 16.85p = 0.0273; nMSE = 0.84p = 0.0273). The most important brain regions predicting borderline traits included the cerebellar regions, parietal areas, the Heschl area, frontal areas, the thalamus, the cingulum, and the insula (see Table 2 for a complete list and Fig. 3). Brain plots were generated with SurfICE software (https://github.com/neurolabusc/surf-ice).

**Table 2:** Brain areas emerged in the whole brain analysis on borderline traits.

ROI label	ROI weight (%)	ROI size (vox)	Exp. Ranking
Cerebelum_7b_R	1.9958	692	113
Angular_L	1.8370	2739	112.8000
Angular_R	1.8005	3628	112.8000
Cingulum_Post_L	1.7485	1094	111
Vermis_9	1.7029	388	97.4000
Cerebelum_Crus2_R	1.6964	3901	109
Heschl_L	1.6802	549	100.2000
Cerebelum_8_L	1.6438	2619	106.8000
Parietal_Sup_R	1.5234	3557	106.8000
Rectus_L	1.4887	1780	105.8000
Heschl_R	1.4100	513	104.2000
Cerebelum_7b_L	1.3940	863	90.2000
Cingulum_Post_R	1.3609	763	98.6000
Precuneus_R	1.3327	7251	96.6000
Frontal_Inf_Oper_R	1.3172	2838	95.4000
Vermis_8	1.3060	528	90.8000
Cerebelum_6_R	1.2940	4096	89.6000
Precuneus_L	1.2631	7574	103
Rectus_R	1.2184	1571	96
Thalamus_L	1.1852	2420	86.6000
Insula_L	1.1510	4518	87
Supp_Motor_Area_L	1.1336	4720	96.8000
Parietal_Inf_R	1.1331	2671	84.8000
Cerebelum_Crus2_L	1.1302	4105	79.8000
Cuneus_L	1.1254	3484	79.2000
Postcentral_R	1.1191	6986	86.6000
Cerebelum_9_L	1.1106	1407	76.4000
Cerebelum_Crus1_R	1.1075	4791	81.8000
Fusiform_R	1.0916	5731	83.2000
Frontal_Mid_Orb_L	1.0833	1210	83.4000
Insula_R	1.0671	4160	84.2000
Precentral_R	1.0612	6310	88.8000
Lingual_R	1.0473	5574	84.4000
Temporal_Sup_L	1.0357	5312	71.8000
Parietal_Inf_L	1.0226	5610	82
Temporal_Inf_R	1.0170	7209	76.8000
Cuneus_R	1.0059	3363	78

*Note.* ROI labels are derived from the AAL atlas. Please note that only regions whose contribution exceeded the 1% are displayed.

### 3.2 A predictive model of borderline traits based on a BPD classification circuit

Using the BPD circuit from Grecucci et al. (2022) as a second-level mask significant results were found for borderline traits (r = 0.12p = 0.033; R2 = 0.01p = 0.6138; MSE = 16.81p = 0.0247; nMSE = 0.84p = 0.0247). The most weighted brain regions predicting borderline traits included the cingulate gyrus, the Heschl area, the parietal areas, and the thalamus (see Table 3 for the completed list).

**Table 3:** Brain areas emerged using a BPD mask on borderline traist.

ROI label	ROI weight (%)	ROI size (vox)	Exp. Ranking
Cingulum_Post_R	12.0282	763	112.4000
Heschl_L	10.2591	549	110.4000
Parietal_Sup_R	9.7853	3557	111.4000
Thalamus_L	8.2674	2420	109.4000
SupraMarginal_R	7.4618	3768	109.4000
Fusiform_R	6.1190	5731	107
Parietal_Sup_L	5.6546	4364	107.6000
Vermis_7	5.6459	458	103.2000
Lingual_R	5.1422	5574	105
Occipital_Mid_R	4.9205	4649	105.6000
Fusiform_L	4.2699	5282	103.8000
Temporal_Mid_L	4.2632	11,409	102.8000
Frontal_Inf_Orb_L	3.8422	4083	100.4000
Frontal_Mid_Orb_R	3.8395	1583	100.4000
Frontal_Sup_Orb_R	3.0106	1352	100
Pallidum_L	2.4882	637	98.6000
Putamen_R	1.7269	2560	96.6000
Amygdala_R	1.2757	571	97

### 3.3 Macro-networks prediction for borderline personality traits

**Fig. 4:**
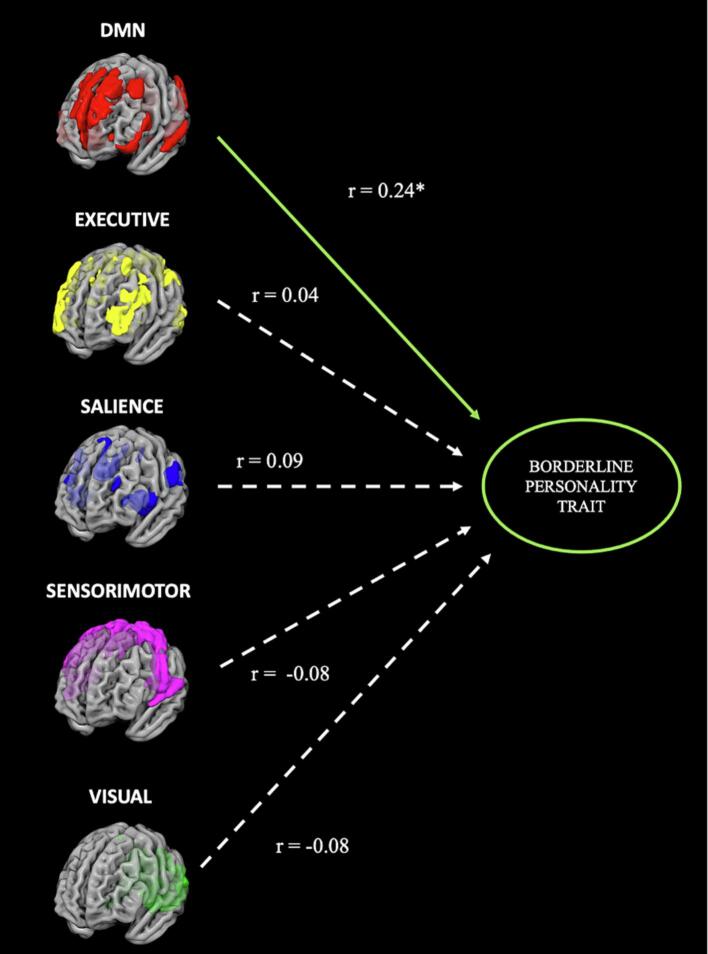
Results for significant networks predicting borderline trait. Results marked with * indicate a significant p-value after Bonferroni correction. Only the DMN successfully predicted borderline traits.

After Bonferroni correction, the default mode network significantly predicted borderline traits (r = 0.24, p = 0.004; R2 = 0.06p = 0.0197; MSE = 16.10p = 0.0033; nMSE = 0.80p = 0.0033). For the salient and the central executive network, a positive association was found which turned out to be non-significant after Bonferroni correction (SN: r = 0.04, p = 0.093; R2 = 0.00p = 0.897; MSE = 17.14p = 0.0733; nMSE = 0.86p = 0.86; CEN: r = 0.09, p = 0.054; R2 = 0.01p = 0.7181; MSE = 17.04p = 0.0536; nMSE = 0.85p = 0.0536). For the sensorimotor and the visual network, no positive association was found (sensorimotor: r = − 0.08; R2 = 0.01; MSE = 18.67; nMSE = 0.93; visual: r = − 0.03; R2 = 0.0; MSE = 17.44; nMSE = 0.87) (For an overview see Fig. 4). Within the DMN network, the most important brain regions predicting borderline traits included cerebellar regions, frontal areas, temporal areas, and the insula (see Table 4 for the complete list and Fig. 4 for a schematic overview).

**Table 4:** Brain areas emerged with the default network mask analysis on the borderline trait.

ROI label	ROI weight (%)	ROI size (vox)	Exp. Ranking
Cerebelum_4_5_R	5.1619	13	110
Cingulum_Ant_L	3.7638	16	109.6000
Frontal_Inf_Tri_L	3.7459	20	107.6000
Fusiform_R	3.4166	131	104.6000
Temporal_Inf_R	3.3483	37	106.4000
Insula_R	3.1627	56	106.2000
Supp_Motor_Area_R	2.9658	335	105.8000
ParaHippocampal_R	2.8484	13	99.6000
Frontal_Inf_Orb_L	2.7010	127	102.4000
Frontal_Mid_Orb_L	2.5024	262	101
Rectus_R	2.3831	190	98.4000
Postcentral_L	2.2882	11	93.4000
Temporal_Sup_R	2.1199	16	96.2000
Insula_L	2.0395	175	92
Hippocampus_L	1.9174	196	93.2000
Cerebelum_Crus1_L	1.8993	2	102
Calcarine_R	1.8865	299	89.2000
Precuneus_L	1.8414	993	90.8000
Cingulum_Post_R	1.8227	441	91.4000
Temporal_Mid_L	1.7680	2	83.8000
Precuneus_R	1.7283	1221	89.4000
Cingulum_Mid_L	1.7268	645	90
Angular_R	1.6656	941	87.6000
Temporal_Pole_Mid_L	1.6092	1146	89.2000
Frontal_Inf_Oper_L	1.4981	18	81
Lingual_R	1.4934	48	78.2000
Cuneus_R	1.4807	225	78
Cingulum_Mid_R	1.4566	346	82.6000
Frontal_Sup_Orb_L	1.4565	1015	83
Olfactory_L	1.4312	274	89.6000
Frontal_Inf_Orb_R	1.3223	829	79.6000
Temporal_Pole_Sup_L	1.3120	56	79
Cingulum_Post_L	1.2811	527	77.6000
Vermis_6	1.2493	5	75.2000
Frontal_Sup_Medial_R	1.2354	1639	75
Occipital_Sup_R	1.2007	18	69.4000
Frontal_Mid_Orb_R	1.1887	391	74.6000
Temporal_Pole_Sup_R	1.1769	20	77.2000
Frontal_Mid_Orb_R	1.1523	51	70.6000
Parietal_Inf_R	1.1511	388	72.2000
Paracentral_Lobule_L	1.1476	1218	73.6000
Frontal_Sup_Orb_R	1.1209	46	71.6000
Frontal_Sup_R	1.1069	1111	73
Frontal_Mid_Orb_L	1.0445	1246	70.6000
SupraMarginal_L	1.0340	213	69.8000
Lingual_L	1.0223	93	68.6000

*Note*. ROI labels are derived from the AAL atlas. Please note that only regions whose contribution exceeded the 1% are displayed.

### 3.4 A predictive model of histrionic traits based on the borderline personality traits circuit and whole brain

Using the extracted borderline traits circuit of Aim 1 as a second-level mask, no significant results were found for histrionic traits (r = 0.00; r^2^ = 0.00; MSE = 20.00; nMSE = 0.87). Moreover, trying to predict histrionic traits from the whole brain (without a BP mask), no significant results were obtained (r = -0.10; r^2^ = 0.01; MSE = 23.61; nMSE = 1.03).

## 4 Discussion

Although borderline personality disorder is the most frequently diagnosed personality disorder, and its neural bases have been recently explored, we still do not know whether the same brain abnormalities that characterize BPD also characterize BPT in a subclinical population. According to recent developments in the diagnostic systems (ICD-11 and DSM-5) personality disorders span over a continuum of traits severity (dimensional view of personality disorders). In the present study, following this dimensional approach we aimed to predict borderline personality traits in a subclinical population from brain structural features to test several hypotheses. First, we tested the hypothesis that BPT can be predicted from whole brain features (Aim 1). As such, this analysis can shed light on the neural bases of borderline personality traits. Then, we tested the hypothesis that BPT prediction can be improved when considering the regions limited to a circuit previously found to correctly classify BPD from HC. This was specifically done to provide eventual evidence of an overlap between clinical and subclinical borderline traits. Next, we tested the possibility to extend the triple network hypothesis of BPD to BPT according to which BPD is characterized by abnormalities in one or more macro networks (DMN, SN, CEN). Last, but not least we tested the hypothesis of a possible overlap between borderline and histrionic personality traits at the neural level. To address these questions, we applied for the first time a supervised machine learning method known as Kernel Ridge Regression to the MRI images of 135 participants. We found that a neural circuit including frontal and parietal areas, as well as the Heschl area, cerebellar regions, the thalamus, the cingulum, and the insula predicted BPT. This prediction was also possible, and slightly statistically improved (higher regression value, and lower p-value), when considering only the regions included in the neural circuit previously found for BPD. The DMN was the only macro-network able to correctly predict BPT. We could not predict histrionic traits from the same brain circuit predicting BP traits. In the next sections, we describe these results in detail.

### 4.1 The neural bases of borderline personality traits

The first result of our study is that we could predict borderline personality traits as measured by the PSSI when considering the whole brain. Our model shows that the most important structural brain area in the sub-clinical borderline seems to be the cerebellum, located at the back of our brain. In addition to a reduction in gray matter in the cerebellum of patients with BPD, Cao et al. (2022) found also a decrease in the right MCG, left IFG, left SFG, and right insula compared to patients with depression. Supporting the unstable pattern of affective regulation in BPD patients, De Vidovich et al. (2016) found a significantly worse performance on an affective Go/No-Go task in BPD compared to healthy controls. Moreover, using rTMS on the left cerebellum, the researchers were able to increase the BPD performance to the level of healthy controls. From these findings, the authors hypothesized that emotional dysregulation and poor impulse control are caused by disrupted cerebello-thalamic-cortical connection in BPD patients (De Vidovich et al., 2016). Our results, along with the above-mentioned findings, suggest that a structural change in the cerebellum might contribute to the symptoms of affective dysregulation in BPD patients. Supporting our insights, Grecucci et al. (2022) also found that the cerebellum was one of the larger contributions for distinguishing between BPD and HC. These results suggest that the cerebellum seems to be involved in the development of BPD, but its role is not clear yet.

The second most important area for predicting borderline traits in our model is the angular gyrus, located at the junction of the occipital, temporal, and parietal lobes. Our model shows bilateral involvement of the angular gyrus. The angular gyrus is described as a cross-modal hub that combines and integrates multiple sensory information to understand and provide meaning to events, manipulate mental representations, and redirect attention to salient information (Seghier, 2013). In a study on self vs other representation, researchers found that a) BPD patients displayed less integration of components of the self, fewer clearly defined boundaries between self and others, and worse maintenance of one’s own and other’s personality representations, and b) a hyperactivation in brain areas comprising the right angular gyrus, the mPFC and the precuneus in comparison to a control group (Beeney et al., 2016). Consistently, Ruby and Decety (2004) found a cluster in the right angular gyrus and the cingulate gyrus crucial for the self-other distinction process. Furthermore, Minzenberg et al. (2008) found lower gray matter concentration in the left anterior cingulate gyrus, supporting our findings of the contribution of the cingulate in our model. These and our findings indicate that the angular and cingulate gyri play a key role in social cognition and self-perception, which are both neglected in BPD.

Regarding subcortical structures, the thalamus seems to play a key role in patients with BPD. Prior studies have reported structural changes to the thalamus in a variety of psychiatric disorders (Hibar et al., 2016, van Haren et al., 2016). In their study, Nenadić and colleagues focused on borderline patients, discovering a link between the intensity of the symptoms and the GM concentration of the left thalamus (Nenadić et al., 2020). Our findings support a structural GM change in the thalamus of BPD patients. The thalamus is associated with general mental functions including attention, memory, and consciousness (Ward, 2013), in addition to emotion (Arend et al., 2015) and reward (Komura et al., 2001). This underpins our hypothesis of thalamic involvement in the traits circuit and its association with emotional dysregulation and impulsivity in BPD patients.

The OFC is another crucial region in BPD neuroimaging research (van Elst et al., 2003). The OFC connects to cortical and subcortical limbic areas and regulates emotional reactions as well as action inhibition (Rolls, 2019). Similar to our findings, other studies have discovered a reduction in GM volume in the medial OFC for BPD in comparison to controls (van Elst et al., 2003). Moreover, it appears that the OFC influences anger and its regulation (Sorella et al., 2021), which are both dysregulated in BPD patients. Lee et al. (2011) found that gray matter volume in the lateral OFC is negatively correlated with impulsivity in psychiatric patients.

Different from other findings (Grecucci et al., 2022, Sampedro et al., 2021) our model did not include the amygdala as one of the main contributors to the prediction. This could be due to the fact that we have analyzed a subclinical population, in which the emotion dysregulation level may not be so exacerbated and thus the amygdala is not structurally compromised as previous studies on BPD showed (Grecucci et al., 2022).

Of note, when considering the regions of the brain limited to the circuit previously found to classify BPD from HC (Aim 2), the performance of the model slightly improved (in terms of regression value and p-value). This further supports the idea of personality disorders as lying on a continuum of trait severity. The same circuit affected in BPD is also affected in BPT and can be used to predict BPT in a subclinical population.

### 4.2 Default mode network contribution to borderline traits

When decomposing the whole brain into the five macro networks, only the DMN was able to predict borderline traits. The DMN is known to be activated during rest and deactivated during cognitive processing of stimuli and is associated with anxiety, rumination, and other pathologies (Vicentini et al., 2017, Zhou et al., 2020). Associated brain areas, such as the medial prefrontal cortex, the posterior cingulate cortex, and the posterior parietal cortex, form an interconnected system that is involved in self-related cognitive activity like social functions and self-monitoring (Bear et al., 2016, Menon, 2011). Furthermore, the angular gyrus represents a core part of the DMN (Smallwood et al., 2021) as well as the OFC (Greicius et al., 2003). Our model shows that the most weighted areas within this network include the cerebellum, the cingulate gyrus, frontal and temporal areas, as well as the hippocampus, confirming some of the findings of the whole brain prediction (Aim 1), and the BPD circuit prediction (Aim 2). In line with our results, Yang et al. (2016) found enlarged GM volume in the main knots of the DMN. Additionally, fMRI studies have confirmed abnormal functional connectivity within the DMN (O’Neill et al., 2015, Wolf et al., 2011) in BPD patients. Our results support the hypothesis that BPD is associated with an impairment in the DMN, which may account for intensive processing of internal thoughts and a dysregulation of emotions during social cognition. We could not find any support for the involvement of SN and CEN in BPT in our study. Although previous studies (Doll et al., 2013) showed the involvement of the triple network in BPD, this may be something specific to a clinical rather than a subclinical population. Probably, the abnormalities in these networks are somewhat not yet detectable in a subclinical population. Of note, the SN and CEN display a mild trend toward significance (without considering the Bonferroni corrected threshold that we adopted to control for multiple comparisons). One possibility is that SN and CEN may reach significance if the traits considered are higher.

### 4.3 Borderline traits do not overlap with histrionic traits at the neural level

Additionally, we investigated the possibility of predicting histrionic traits from the circuit that predicts borderline traits derived from Aim 1. If this is true this would mean that the two personality traits, borderline and histrionic, share not only symptoms but also at least some neural mechanisms. If this is false, this may be interpreted as borderline and histrionic to be different from a neural point of view, even though they share some clinical features. We found evidence of this second possibility. The negative result could be interpreted to mean that HP does not overlap with BP at least at a neural level.

## 5 Conclusion

In the present study, we employed supervised machine learning (KRR) to investigate the hypothesis that borderline traits can be predicted based on specific structural brain features in a subclinical sample. As we discovered similar brain regions including the cerebellum, thalamus, and frontal areas related to borderline personality disorder (Grecucci et al., 2022), our findings account for the existence of a continuum between subclinical borderline traits and borderline personality disorder. Our statistical approach contributes to the development of possible biomarkers for BPD and may be used to identify new, unobserved cases. It is thus a reliable diagnostic tool for use in clinical settings. Furthermore, we provided early evidence of the specificity of such borderline biomarkers, that predicted borderline personality but not histrionic personalities (despite the overlapping at the behavioral level). Since we were incapable of creating such a model, these two disorders should likely be treated independently. Finally, we tried to develop a model to identify brain areas that are predictive of histrionic traits. We were unable to develop such a model, possibly because histrionic traits are not associated with any particular brain regions. Therefore, our research contributes to resolving the controversy surrounding the classification of personality disorders.

## 6 Limitations

Despite the merits, our study does not come without some limitations.

First, we only included gray matter in our model, excluding the possibility of white matter or functional brain features serving as biomarkers. It may be worthwhile to investigate this, as histrionic traits could be predicted by white matter and thus contributing to a better understanding of the neural basis of this trait.

Second, although the use of a subclinical sample includes many advantages, such as no drug consumption and a larger sample size, it could represent a limit for some aspects. In particular, in the subclinical sample borderline is measured as a dimensional personality trait which is different to the categorical classification of borderline disorder. Although the current diagnostic manuals consider the dimensional approach, the patients from which Grecucci et al. (2022) used the structural images were diagnosed based on the purely categorical system. This leads to the constraint that our trait results cannot be equated with previous studies on BPD. Furthermore, a progressed borderline disease could be reflected in stronger structural brain changes which are not detectable in a subclinical sample. Moreover, the subclinical sample scored rather low and skewed on the borderline trait. Therefore, it might be not advisable to base the use of biomarkers only on a subclinical brain circuit. An additional limitation is that data on cognitive and emotional factors was not available. Therefore, it was not possible to include these factors as possible covariates that might mediate the relationship between borderline personality traits and the observed networks. Future research should aim to collect more clear data.

Lastly, the negative results regarding histrionic traits could be partly due to the minimal prior research on these traits. In contrast to HP traits BPD and borderline traits are widely investigated and therefore well understood. Regarding our results future research should not neglect histrionic traits but further investigate this area and consider the discussed diagnostic manuals critically.

## Declaration of Competing Interest

Author SB was funded by the European Union’s Horizon 2020 Framework Programme for Research and Innovation under the Specific Grant Agreement No. 945539 (Human Brain Project SGA3). The authors declare that they have no known competing financial interests or personal relationships that could have appeared to influence the work reported in this paper.

## Data Availability

Data was used from the open data set: OpenNeuro database, Accession Number: ds000221. This data was conducted by the Max Planck Institute (MPI) of Human Cognitive and Brain Sciences in Leipzig.
